# Budding Yeast: An Ideal Backdrop for *In vivo* Lipid Biochemistry

**DOI:** 10.3389/fcell.2016.00156

**Published:** 2017-01-10

**Authors:** Pushpendra Singh

**Affiliations:** ^1^Center for Cell Dynamics, Department of Cell Biology, Johns Hopkins University School of MedicineBaltimore, MD, USA; ^2^Department of Chemical and Biomolecular Engineering, Whiting School of Engineering, Johns Hopkins UniversityBaltimore, MD, USA

**Keywords:** budding yeast, lipid-protein interactions, lipid sensors, sphingolipid, ergosterol

## Abstract

Biological membranes are non-covalent assembly of lipids and proteins. Lipids play critical role in determining membrane physical properties and regulate the function of membrane associated proteins. Budding yeast *Saccharomyces cerevisiae* offers an exceptional advantage to understand the lipid-protein interactions since lipid metabolism and homeostasis are relatively simple and well characterized as compared to other eukaryotes. In addition, a vast array of genetic and cell biological tools are available to determine and understand the role of a particular lipid in various lipid metabolic disorders. Budding yeast has been instrumental in delineating mechanisms related to lipid metabolism, trafficking and their localization in different subcellular compartments at various cell cycle stages. Further, availability of tools and enormous potential for the development of useful reagents and novel technologies to localize a particular lipid in different subcellular compartments in yeast makes it a formidable system to carry out lipid biology. Taken together, yeast provides an outstanding backdrop to characterize lipid metabolic changes under various physiological conditions.

## Introduction

Plasma membrane outlines the boundary of a living cell by separating it from the environment, thus provide it protection and identity. Compromising membrane integrity adversely affects the cellular function leading to release of calcium and local accumulation of vesicles (Krause et al., 1994). It has been proposed that increased membrane tearing results in cell death due to overwhelming repair process (Petrof et al., 1993; McNeil and Steinhardt, 1997). Plasma membrane acts as a selective barrier as well as means of communication with extracellular environment through signal transduction in a cell (Harder, 2012; Astro and de Curtis, 2015). In addition, membranes compartmentalize eukaryotic cell into different subcellular structures and act as scaffold for certain enzymatic reactions that allow reactions to be spatially confined inside a cell and 3-D cytosol to 2D membrane (Dislich and Lichtenthaler, 2012), respectively.

Biological membranes are non-covalent assembly of phospholipids, sterols and proteins. About 20–30% of eukaryotic genome has been estimated to encode for the membrane proteins (Krogh et al., 2001; Almen et al., 2009). Phospholipid species could be categorized into thousands types in eukaryotic cells based on their head group, acyl chain length and number and position of double bonds in it (Fahy et al., 2009). Because of their small size and hydrophobicity, lipids exhibit constant lateral and transverse movements in membrane bilayer providing it fluid-like characteristics whereas mobility of membrane proteins is somewhat restricted. Membrane proteins transduce information across the bilayer, thus establish communication to external environment. Transmembrane domain of proteins are embedded in membrane and thus interact with lipids in bilayer. Lipids have been shown to regulate function of different membrane associated proteins either directly through modulating their functions (Contreras et al., 2011; Laganowsky et al., 2014) or indirectly by altering physical properties of membrane bilayer (Lee, 2004; Lundbaek et al., 2010). Numerous membrane proteins have been found to exhibit specificity toward certain lipid for their organization and function (Contreras et al., 2011; Laganowsky et al., 2014). A detailed information of lipid environment around a protein is therefore required to understand the function of cell membranes and membrane proteins (Coskun and Simons, 2011).

Lipids and proteins self-assemble through non-covalent interactions in biological membranes where certain lipids such as cholesterol and sphingolipids are known to exhibit higher mutual affinity leading to ordered domain formation termed as “lipid raft.” Such membrane domains are believed to be important for various cellular processes such as signal transduction, membrane trafficking in mammalian cells (Simons and Ikonen, 1997; Simons and Toomre, 2000; Simons and Vaz, 2004; Simons and Sampaio, 2011). In order to understand the organization of lipids and proteins and lipids' structural specificity in determining protein function in such domains, model membranes such as small unilamellar vesicles (SUVs), large unilamellar vesicles (LUVs), giant unilamellar vesicles (GUVs), supported lipid bilayers (SLBs) and biochemical approaches were employed (Smith, 2012; Zhao and Lappalainen, 2012; Lagny and Bassereau, 2015). However, studies in these systems are challenging due to limited success in membrane protein purification and their poor reconstitution in desired lipid environment (Seddon et al., 2004). Moreover, they do not provide systems-level understanding of highly-complex biological membranes owing to presence of limited diversity in lipid composition.

Biochemical approaches were invented to understand the organization and distribution of lipids and proteins in biological membranes based on differential detergent solubility (Brown and Rose, 1992; Lichtenberg et al., 2005) and differential fractionation on sucrose density gradient (Yao et al., 2009) of membrane domains. Detergent resistant membranes (DRMs) were speculated to be equivalent to “lipid raft.” However, lipid-protein composition of such domains varied depending on the method of isolation (Lichtenberg et al., 2005; Babiychuk and Draeger, 2006; Williamson et al., 2010). In fact, detergents itself were shown to induce domain formation rather isolation of naturally existing membrane domains (Heerklotz, 2002). Knowledge gained from these systems therefore remain dubious and do not provide the real picture of lipid-protein distribution and function in a cell. To gain systems-level understanding of the aforementioned issue, a biological system, carrying adequate complexity, yet amenable for lipid composition manipulation is required.

## Yeast: an ideal system for lipid-protein interactions

Budding yeast *Saccharomyces cerevisiae* is a powerful and convenient model organism for research in cell and membrane biology. It offers a unique advantage to understand the lipid-protein interactions due to availability of a vast array of genetic and cell biological tools. *S. cerevisiae* is genetically tractable and has benefitted almost every discipline of biology in general and cell biology in particular. A large collection of tools e.g., genome-wide yeast strain libraries carrying open reading frame (ORF) deletions (Winzeler et al., 1999; Giaever et al., 2002), genes tagged with high-affinity epitope for biochemical protein purification (Puig et al., 2001; Ghaemmaghami et al., 2003) or GFP (Huh et al., 2003) are available for budding yeast. In addition, synthetic genetic array (SGA) strategies (Baryshnikova et al., 2010a,b; Costanzo et al., 2010; Wagih et al., 2013; Chong et al., 2015), allow to study potential genetic interactions among genes in different pathways. These genomic collections are very useful for characterization of genes and proteins involved in lipid metabolism. Importantly, genome of *S. cerevisiae* is annotated thoroughly (Goffeau et al., 1996) that allows the identification of gene/protein homologs in human and other eukaryotes (Zhang and Bilsland, 2011), therefore enabling the application of knowledge gained in yeast to higher mammals including human.

As a model system, yeast offers several additional advantages for comprehensive understanding of lipid biology. Yeast can be cultured in completely defined media under simple and controlled growth conditions allowing an accurate interpretation of lipid associated phenotype as opposed to mammalian cells which are generally grown in serum containing medium. Serum is the rich source of lipids and fatty acids besides growth factors and other nutrients, therefore interpretation of lipid associated defects is difficult in mammalian cells under such conditions. Importantly, lipid metabolic pathways are well conserved between yeast and other eukaryotes (Lykidis, 2007; Hannich et al., 2011). Yeast has relatively simple repertoire of lipids in the range of several hundred (Guan and Wenk, 2006; Ejsing et al., 2009) compared to thousands of lipid species in mammalian cells (Yetukuri et al., 2008; Sampaio et al., 2011). Taken together, robust information can be generated in greater detail in budding yeast in a relatively short span of time due to its shorter doubling time, simple lipid metabolic pathways and well characterized genome.

## Lipid homeostasis in yeast

Biosynthesis and metabolism of glycerophospholipids, sphingolipid, and sterols in yeast have been discussed extensively in literature (Dickson, 2008; Carman and Han, 2009; Hannich et al., 2011). Lipid metabolism pathways in yeast are simpler as compared to mammalian cells, given the higher number of genes with multiple paralogs as suggested by complexity of mammalian lipidome (Quehenberger and Dennis, 2011; Sampaio et al., 2011) yet core lipid biosynthetic pathways are conserved from yeast to human (Kurat et al., 2006; Nielsen, 2009). Yeast has been instrumental in the discovery and characterization of many genes involved in lipid metabolism. Yeast deletion collections has been employed in number of high-throughput screens to investigate the phenotype of gene deletion and its interactions with other genes in lipid metabolism. For example, systematic analysis of yeast strains revealed genes that cause defect in lipid metabolism (Daum et al., 1999). In addition, a genome wide screen helped reveal the role of sphingolipids and ergosterol to cell surface delivery, identification of inositol auxotrophic phenotypes (Hancock et al., 2006; Villa-Garcia et al., 2011) and genes responsible for lipid droplet formation (Szymanski et al., 2007; Fei et al., 2008; Bozaquel-Morais et al., 2010). In order to gain insights about the spatial localization of lipid biosynthesis, green fluorescent protein (GFP) collection of yeast strains was harnessed. By surveying localization of GFP tagged enzymes of lipid biosynthesis, it was observed that ER is the main organelle for lipid synthesis. In addition, significant number of these enzymes were observed in mitochondria, Golgi vacuoles, and vesicles (Natter et al., 2005). Genetic approaches have tremendously helped discovery and functional characterization of genes involved in lipid metabolism.

## Organization and dynamics of lipids and proteins in yeast plasma membrane

Plasma membrane in mammalian cell usually contains, ~30–40% cholesterol and ~10–20% sphingolipid of total plasma membrane lipids (Lange et al., 1989; van Meer, 1989). Budding yeast does not have cholesterol and sphingomyelin instead contains inositol phosphoceramide (IPC) and ergosterol, an equivalent of mammalian sphingolipid and cholesterol (Montefusco et al., 2013, 2014; Aguilera-Romero et al., 2014). As mentioned earlier, cholesterol and sphingolipids are known to form ordered domains which are believed to be important for various cellular processes (Simons and Ikonen, 1997; Simons and Toomre, 2000; Simons and Vaz, 2004; Simons and Sampaio, 2011). Similar domains are observed in budding yeast where they are enriched in ergosterol and complex sphingolipids (Kubler et al., 1996; Bagnat et al., 2000) including IPC, mannose-inositol-phosphoceramide (MIPC), and mannose (inositol phosphate) 2-ceramide (M(IP)2C) (Dickson et al., 2006; Dickson, 2008). Such ordered domains are also known as membrane compartment of Can1 (MCC) and membrane compartment of Pma1 (MCP, Malinska et al., 2004; Grossmann et al., 2007), eisosomes (Walther et al., 2006) in yeast. Later studies revealed that yeast plasma membrane is rather domainized (Spira et al., 2012) probably due to inherent slow diffusion of lipids (Greenberg and Axelrod, 1993) and proteins (Valdez-Taubas and Pelham, 2003) in yeast plasma membrane. Interestingly, yeast plasma membrane were observed to slow down the lateral diffusion of heterologous expressed human serotonin_1A_ receptor as compared to that in mammalian cells (Ganguly et al., 2009).

## Lipid visualization methods

Research of decades has enhanced our understanding about lipids' functions and establish them as active membrane components (Watkins et al., 2011). Presence of specialized membrane domains such as lipid rafts are proposed to be hub for cellular signaling, membrane sorting, and endocytosis reviewed in (Simons and Ikonen, 1997; Simons and Toomre, 2000; Simons and Vaz, 2004; Simons and Sampaio, 2011). However, existence of lipid rafts still remains a matter of debate in cell biology. In addition, organization and dynamics of lipids in membranes of different subcellular structures have not been probed accurately. Interestingly, dynamics of lipid metabolism is altered during cell cycle progression in mammalian fibroblast cells (Singh et al., 2013) and aging (Choi et al., 2015). For example, about 40% increase was observed in cholesterol content in rat fibroblast cells (Singh et al., 2013) while sphingolipid levels are dysregulated during aging rats and mice (Sacket et al., 2009; Babenko and Shakhova, 2014; Mc Auley and Mooney, 2015).

Visualization of lipids in native environment has been challenging due to limited availability of appropriate probes to recognize naturally occurring lipids in live cell. Novel tools are being developed to investigate the localization and dynamics of lipids in different subcellular compartments in live cell (Maekawa and Fairn, 2014). Based on their mode of incorporation in cell membrane, lipid probes can be categorized as exogenous and endogenous. Exogenous lipid probes are fluorescently tagged lipid analogs, antibodies and lipid binding protein domains that get incorporated in cell membrane upon exogenous supplementation. Lipid probes used to study lipid domains comprise analogs of sterols such as cholestatrienol (Nystrom et al., 2010), dehydroergosterol (DHE) and 25-NBD-cholesterol (Wustner, 2007), phospholipids (Eggeling et al., 2009) and fluorescent tagged proteins in mammalian cells (Wustner, 2007). High resolution microscopy using fluorescence correlation with fluorescently labeled lipids has demonstrated that lipid diffusion is restricted in certain domains of the plasma membrane (Eggeling et al., 2009). Exogenous lipid probes are useful and get readily incorporated in the membrane, but may not be a good mimic of naturally occurring lipids, as observed for the cholesterol fluorescent analog dehydroergosterol (DHE) and 25-NBD-cholesterol. DHE preferentially localizes in liquid ordered domain whereas 25-NBD-cholesterol majorly partitions in disordered domains (Wustner, 2007).

Lipid-binding proteins, such as lysenin (Ishitsuka and Kobayashi, 2004), cholera toxin (Heyningen, 1974), S.V. equinatoxin (Barlic et al., 2004; Yachi et al., 2012) have been useful in studying membrane domains in plasma membrane. These motifs are part of different amphitropic proteins where they help proteins to associate with membranes by binding to unique lipid. Lipid binding toxins need to be improved and evolved to circumvent their harmful effects as they are shown to kill cells by forming pore in plasma membrane. Antibody against lipids is an effective novel approach to visualize lipids in live cell. For example, antibodies against the lyso (bis) phosphatidic acid (LBPA, Kobayashi et al., 1998) phosphatidylglucoside (Murate et al., 2010), ceramides (Cowart et al., 2002), and even an antibody that recognizes ceramide/cholesterol enriched domain have been described (Scheffer et al., 2006). However, generating antibody of high specificity against a lipid is challenging due to their poor antigenicity and highly similar structures.

Further, presence of cell wall around yeast poses a limitation for the usefulness of exogenous lipid probes as opposed to higher eukaryotes. Labeling of plasma membrane can be probably achieved by shaving off cell wall enzymatically. However, it might affect the organization of lipids and proteins in plasma membrane as cell wall interacts with plasma membrane through embedded proteins in it. Importantly, cells are fixed to freeze distribution of lipids and protein and later labeled with fluorescent tagged lipid binding antibodies and toxins. However, fixation methods like formaldehyde and glutaraldehyde does not entirely preserve the localization of integral proteins and lipids (Hammond et al., 2009; Tanaka et al., 2010). This creates a concern for their applicability in determining proper localization of lipids in a cell.

Endogenous probes are genetically encoded fluorescent protein domains of amphitropic protein and toxins that specifically bind to a lipid. They can access lipids in plasma membrane as well as in membranes of subcellular structures therefore provide information about localization and dynamics of different lipids. These probes can be expressed as an epitope-tagged fusion to allow for lipid visualization using a plasmid-based biosensor. Examples of this strategy include the use of the pleckstrin homology (PH) domain from phospholipase C for phosphatidylinositol 4,5-bisphosphate (PIP2) and the Lact-C2 domain for phosphatidylserine (PS) in *S. cerevisiae* (Fairn et al., 2011; Das et al., 2012). In particular, these probes have been successfully used to monitor the localization and distribution of PS and PIP2 in *S. cerevisiae* where PS gets concentrated at polar cortex during cell polarization (Das et al., 2012) NCB whereas distribution of PIP2 remains uniform all over the cell cortex. Endogenous lipid probes provide advantage over exogenous probe as no staining procedure is required. In addition, localization of lipids could be fixed using formaldehyde under these conditions as that can fix protein domain of these probes. However, endogenous lipid probes recognize “free” lipids and may sequester these lipids if overexpressed in a cell thus making lipid molecule unavailable for their physiological function. Budding yeast offers tremendous potential for the development of these probes because of its facile genetics. Employing yeast, new tools are being developed to probe the distribution of native lipids in the cells by developing specific antibodies against different lipids. For example, attempts are in place to develop bicyclic peptides to specifically bind sphingolipids (Heinis et al., 2009; Takahashi-Umebayashi et al., 2011). A systematic screen was targeted to reveal lipid-protein interactions in *S. cerevisiae* (Gallego et al., 2010). For which, nitrocellulose arrays containing different sets of lipids were used to determine the binding profiles of different soluble proteins. They reported several novel surprising interactions indicating that there is a still huge gap in our understanding of lipid-proteins interactions. Such studies provide a starting point for validation of these interactions with newly developed tools.

## Unique reagents in yeast for lipid-protein interactions

In addition to number of available libraries, novel strategies and tools have been developed to study structural importance of different lipids by tweaking structure of lipids through metabolic engineering in yeast. For example, acyl chain remodeling of phospholipids in cardiolipin, phosphatidylcholine, phosphatidylinositol, and phosphatidylethanolamine can be achieved by deletion and overexpression of certain enzymes in yeast reviewed in Renne et al. (2015). These strategies could help in understanding the function of acyl chain remodeling in yeast physiology such as growth, mating and aging. In addition, trafficking of a subset of yeast plasma membrane proteins is known to be dependent on phospholipid and sterol biosynthesis reviewed in Bankaitis et al. (2012). In this regard, engineered yeast strains that produce lipids carrying specific alterations would be useful tool. For example, yeast lipidome has ergosterol and sphingolipids as two major lipids which considerably differ from their equivalents in mammalian cells. Recently, strains have been engineered that produce sphingolipids of shorter chain C18 (Cerantola et al., 2007; Epstein et al., 2012) instead of C26 and cholesterol instead of ergosterol (Souza et al., 2011). These strains would be valuable tool to study the lipid specificity in trafficking and function of membrane proteins and would help in delineating the functional differences between cholesterol and ergosterol on yeast membrane proteins. Lipid homeostasis in yeast is maintained by lipid synthesis and lipid storage. Excess amount of lipids are stored in form of lipid droplets (LDs) thus LDs act as reservoir for membrane components and source of energy during adverse condition. Yeast strains have been constructed that are devoid of lipid storage (lipid droplets) (Sandager et al., 2002). They would therefore be an asset in gaining comprehensive understanding about the role of LDs in cell survival under various physiological and stress conditions and would provide mechanistic details about lipid homeostasis and metabolism.

## Conclusion and future perspective

**Figure 1:**
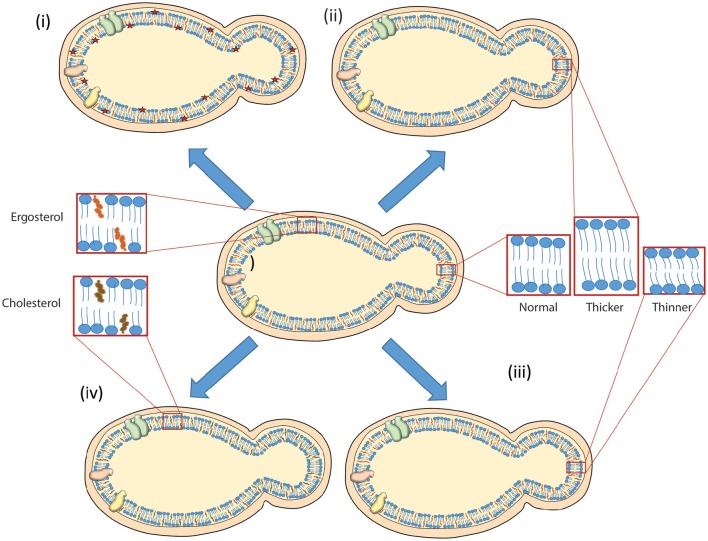
**Scheme depicting different lipid modulations achieved in budding yeast**. A thick cell wall is present around the plasma membrane carrying proteins (shown in cyan, orange, and yellow just to distinguish them as different) in budding yeast. Cell wall and lipids are depicted in orange and blue color, respectively. A handful of examples are presented (i) visualizing lipids with development of fluorescent biosensors as red star (Fairn et al., 2011; Das et al., 2012) (ii) remodeling membrane to be thicker by acyl chain lengthening (Dickson et al., 1990; Gaigg et al., 2006; Renne et al., 2015) (iii) producing thinner membrane by acyl chain shortening (Cerantola et al., 2007; Epstein et al., 2012; Renne et al., 2015) (iv) converting ergosterol to cholesterol in budding yeast (Souza et al., 2011). See text for more details.

Research in yeast have made important contributions to the study of lipid homeostasis and function, and provided significant insights into fundamental pathways in lipid metabolism that could be extended to more complex organisms (Nielsen, 2009). Employing genetic screens and quantification of lipids under different environmental conditions, yeast could help uncover many unsuspected and novel molecular interactions among proteins and lipids. Yeast has potential for the development of new technologies that could help us understand the lipid distribution, interaction and their involvement in biogenesis of different cellular structures and as signaling molecule in cellular signaling events as depicted in Figure 1. Integration of new technologies in budding yeast could help us understand the fundamental questions of aging and diseases. For example, microfluidic chips are being developed to follow the replicative life span in yeast (Zhang and Bilsland, 2011; Jo et al., 2015; Liu et al., 2015), localization of proteins and lipids in yeast could establish the link between dynamics of lipid metabolism with aging. In addition, lipid disorders observed in humans can be replicated in yeast to gain better and robust understanding about their molecular mechanisms that help in development of the treatment against such disorders. Yeast has been employed as model system to find cure for Parkinson's disease (Khurana and Lindquist, 2010). Taken together, yeast would be an ideal system for making advancement in these areas thus providing details regarding the localization of lipids in their native environment under different cellular processes, and enhancing our understanding about the lipid distribution, dynamics and trafficking under different environmental conditions.

## Author contributions

PS collected the material and wrote it.

### Conflict of interest statement

The author declares that the research was conducted in the absence of any commercial or financial relationships that could be construed as a potential conflict of interest.
